# Functional Outcome and Overall Survival in Patients with Primary or Secondary CNS Lymphoma after Surgical Resection vs. Biopsy

**DOI:** 10.3390/cancers15215266

**Published:** 2023-11-02

**Authors:** Franziska Staub-Bartelt, Jos Rittenauer, Michael Sabel, Marion Rapp

**Affiliations:** Department of Neurosurgery, Medical Faculty, Heinrich-Heine University Düsseldorf, Moorenstraße 5, 40225 Düsseldorf, Germanymichael.sabel@med.uni-duesseldorf.de (M.S.); marion.rapp@med.uni-duesseldorf.de (M.R.)

**Keywords:** primary CNS lymphoma, secondary CNS lymphoma, surgical therapy, biopsy, functional outcome, overall survival

## Abstract

**Simple Summary:**

Central nervous system lymphoma is a rarity among brain tumours. The clinical course depends on location and size of the tumour. Various established treatments, including chemotherapy, radiation, and stem cell transplantation, are available. The resection of intracranial lesions is still under discussion. In our retrospective analysis, we were able to show that compared to the sole confirmation of diagnosis through stereotactic biopsy, the resection of the lesion offered a significant advantage in patient survival. Especially for singular, easily accessible lesions, resection in addition to radiotherapy/chemotherapy could have a benefit. Our findings substantiate earlier research outcomes and shall lay the foundation for forthcoming prospective investigations.

**Abstract:**

Background: Central nervous system lymphoma (CNSL) is rare form of brain tumour. It manifests either as primary CNS lymphoma (pCNSL) originating within the central nervous system or as secondary CNS lymphoma (sCNSL), arising as cerebral metastases of systemic lymphoma. For a significant period, surgical resection was considered obsolete due to the favourable response to chemotherapy and the associated risk of postoperative deficits. The objective of the present study was to demonstrate the benefits of resection in CNSL patients, including extended survival and improved postoperative function. Methods: A retrospective study involving patients diagnosed with either PCNSL or SCNSL that were surgically approached at our neurosurgical department between 2010 and 2022 was conducted. Patients were categorised into three subgroups based on their neurosurgical approach: (1) stereotactical biopsy, (2) open biopsy, (3) resection. We then performed statistical analyses to assess overall survival (OS) and progression-free survival (PFS). Additionally, we examined various secondary factors such as functional outcome via Karnofsky Performance Index (KPS) and prognosis scoring. Results: 157 patients diagnosed with PCNSL or SCNSL were enclosed in the study. Of these, 101 underwent stereotactic biopsy, 21 had open biopsy, and 35 underwent resection. Mean age of the cohort was 64.94 years, with majority of patients being female (54.1%). The resection group showed longest OS at 44 months (open biopsy = 13 months, stereotactic biopsy = 9 months). Calculated median follow-up was 34.5 months. In the Cox regression model, postoperative KPS 70% (*p* < 0.001) and resection vs. stereotactic biopsy (*p* = 0.040) were identified as protective factors, whereas older age at diagnosis was identified as a risk factor (*p* < 0.001). In the one-way analysis of variance, differences in postoperative KPS were found among all groups (*p* = 0.021), while there was no difference in preoperative KPS among the groups. Conclusions: Our data show a favourable outcome when resection is compared to either stereotactic or open biopsy. Additionally, the marginally improved postoperative functional status observed in patients who underwent resection, as opposed to in those who underwent biopsy, provides further evidence in favour of the advantages of surgical resection for enhancing neurological deficits.

## 1. Introduction

Central nervous system lymphoma (CNSL) is a rare form of brain tumour, accounting for about 2% of all brain tumours, with an estimated annual incidence of 0.45 to 0.5 per 100,000 individuals in Western countries [1]. This unique tumour entity can manifest as either primary CNS lymphoma (pCNSL), originating within the central nervous system, or secondary CNS lymphoma (sCNSL), which corresponds to the spread of lymphoma from systemic sites to the brain [2,3,4]. The predominant histological subtype of CNSL is Diffuse Large B-Cell Lymphoma (DLBCL) [5]. CNS lymphoma, regardless of its aetiology, can clinically present with a wide range of symptoms. In addition to headaches, nausea, and dizziness, it can also manifest neurological deficits depending on its location. In some cases, it may lead to acute life-threatening situations, such as in the presence of hydrocephalus. The diagnosis of CNSL can be assumed by radiological findings, and even historically implemented stereotactic biopsy still plays an important role in current neuropathological diagnosis [6,7].

As of 2023, there is no universally standardised treatment regimen for CNSL. However, the 2023 European Association of Neuro-Oncology (EANO) guidelines recommend a multi-faceted approach for pCNSL. This includes an induction chemotherapy regimen that incorporates high-dose methotrexate (HD-MTX) along with other immunotherapeutic and chemotherapeutic agents (Level B recommendation). This is followed by consolidation therapy, which may consist of high-dose chemotherapy (HD) combined with autologous stem cell transplantation (ASCT) (Level B recommendation) or radiotherapy (Level A recommendation) [8]. For sCNSL, there is no official guideline, but these entities are also regularly treated by radiation and high-dose chemotherapy, as well as stem cell transplantation [3,9]. Furthermore, there is ongoing research for patients with relapsed pSCNL concerning the best therapy strategies as median survival after relapse is known to be very restricted [10].

In contrary to the consensus on the benefits of radio-chemotherapy for patients with CNSL, the role of surgery for treatment remains a subject of debate and cannot be answered in a standardised way [11]. Surgical resection of CNSL lesions was discouraged due to the favourable response rates to chemotherapy and radiotherapy, which outweighed the potential risks of postoperative deficits. Additionally, many CNSL lesions are multifocal and located within deep brain structures, making surgical removal challenging. However, particularly in cases where lesions might be easy approachable due the localisation or in cases where they might be associated with increased intracranial pressure, resection could be more favourable. There have been previous publications supporting surgical approaches to CNSL [12,13]. According to the latest EANO guidelines, the decision to pursue surgery should be made within an interdisciplinary tumour board, considering the overall clinical context.

Given the evolving landscape of CNSL management and the potential role of surgery in improving patient outcomes, the primary objective of our study was to investigate whether patients diagnosed with CNSL and undergoing neurosurgical resection, according to modern standards, experience extended OS and PFS, along with improved postoperative functional status. Additionally, we aim to discern the factors that may impact survival outcomes within this intricate patient population. To address these critical questions, we conducted a comprehensive retrospective analysis, drawing data from a single centre and encompassing patients treated between 2010 and 2022, all of whom received a neuropathologically confirmed diagnosis of CNSL, encompassing both PCNSL and SCNSL diagnosis.

## 2. Patients and Methods

We here report a single-centre retrospective analysis of 157 CNSL patients who were treated at our neurosurgical department at the University Hospital Düsseldorf, Germany, between 2010 and 2022. Approval of the local ethical committee was obtained beforehand (local study number 2022-1944).

**Figure 1:**
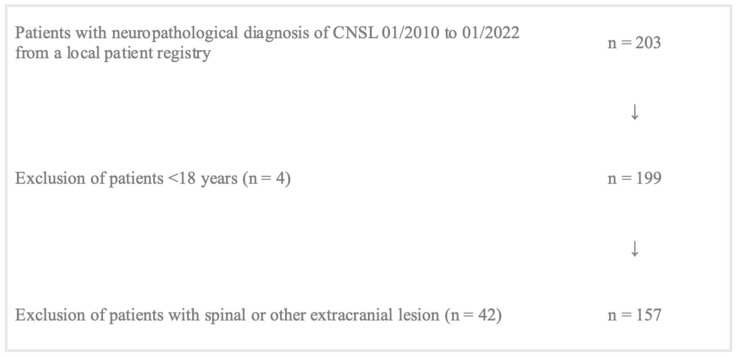
Illustrative flow chart of patient inclusion due to predefined criteria.

Patient data were available from an internal patient registry including patients undergoing any surgical procedure, e.g., resection, open biopsy, or stereotactical biopsy with histopathological outcome of CNSL. In total, 203 patients were screened for inclusion, of whom 157 patients were eligible for analysis. Inclusion criteria were: (1) age > 18 years, (2) at least one cerebral manifestation of lymphoma, (3) histopathologically confirmed diagnosis of CNLS, (4) at least one MRI dataset available. Patients younger than 18 (*n* = 4) and patients with spinal or other extracranial lesions (*n* = 42) were excluded. A flow chart illustrates patient selection for statistical analysis (Figure 1).

In addition to the local patient registry, a source of reported data was the local patient administration system “Medico” (CompuGroupMedical, CGM Clinical Europe GmbH, Koblenz, Germany). The authors included various sociodemographic information such as information on gender and age, as well as information on medical history and results of further diagnostic procedures (lumbar punction) and adjuvant therapies.

### 2.1. Surgical Procedure

In order to evaluate the main outcome of the present study, the impact of surgical approach on progression-free and overall survival of enclosed patients was studied. Therefore, the surgical approach was analysed as a subgroup investigation and thus divided into the following subgroups: (1) stereotactical biopsy, (2) open biopsy including all open approaches for biopsy without aim of resection, (3) resection, meaning all surgical approaches with the aim of resection of the target lesion. Only the target lesion was used for further analysis in cases of multilocular lesions.

### 2.2. Evaluation of Survival

#### 2.2.1. Progression-Free and Overall Survival (PFS and OS)

Patients enclosed in the study were followed up for information on progression-free survival (PFS) and overall survival (OS) until December 2022. Data were obtained from the local patient administration system or via electronic query of the German cancer registry. For calculation of Kaplan–Meier curves, the following events were defined:

OS, event = death, censored = last follow-up;

PFS, event = progress according to RANO criteria in MRI scan or death, censored = last follow-up. Median follow-up was calculated by calculating the survivor function estimate for the observation period among individuals who remained free from the event at the conclusion of the follow-up period [14].

#### 2.2.2. The Memorial Sloan Kettering Cancer Center (MSKCC) Score

The MSKCC Score is a clinical scoring system used to assess prognosis and (median overall) survival outcomes of patients with various types of cancer. Its prognostic value has been well evaluated for patients diagnosed with pCNLS [15,16] and is divided into three classes considering age as well as KPS: class 1 = age < 50, class 2 = age > 50 + KPS > 70, class 3 age > 50 + KPS < 70.

### 2.3. Functional Outcome

For basic information on functional outcome of patients, the Karnofsky Performance Index (KPS) was analysed pre surgical intervention (KPS pre-op) and at dismission (KPS post-op) as this marker was available for all patients enclosed, whereas neurological scoring systems were not available for all patients. Additionally, data were screened for postoperative complications (yes or no) without further detailed description.

### 2.4. Radiological Studies

All MRI scans were obtained from a local radiology information system (SECTRA Workstation 101, IDS7, Version 24.1, Sectra AB, Linkoping, Sweden, 2022). The authors calculated tumour volume in pre-op MRI and post-op MRI using the volumetry tool of the local radiology information system. Furthermore, localisation (supratentorial, periventricular, infratentorial) and number of lesions were analysed (unilocular or multilocular).

### 2.5. Statistical Analyses

Descriptive statistics were evaluated by using crosstab analysis for categorical variables and one-way analysis of variance (ANOVA) to explore variations and differences between groups. For variables that were dichotomous or of ordinal nature, we applied the chi-square test and Fisher’s exact test.

Analysis of continuous-scaled data was performed using one-way ANOVA to investigate variations and differences between groups, particularly when dealing with multiple categories or factors. The Sum of Squares was used to quantify the variability within and between groups, aiding in the determination of the significance of these variations. Furthermore, the t-test was used to compare means between groups. Kaplan–Meier curves were calculated to estimate survival probabilities (PFS and OS). To assess the significance of survival differences between groups, log-rank test was performed. All statistical testing was performed using IBM SPSS Statistics Version 29 for MacOS (IBM Corporation, Armonk, NY, USA). Statistical significance stated as *p*-value for all results was set at 0.05.

For a summary of cohorts’ general data and collected information on prognosis, functional outcome, and survival please refer to Table 1.

## 3. Results

Overall, 157 patients were enclosed in the statistical analyses, of whom 54.1% were female and 45.9% male. Mean age of the cohort at surgery was 64.94 (±SD 12.68). Of these patients, 128 suffered from pCNSL (81.5%) and 29 (18.5%) from sCNSL. Histopathological findings comprised DLBCL; as estimated, this entity comprised the vast majority of diagnosis, with 143 patients (91.1%). As a subgroup, there was Epstein-Barr-related diagnosis of DLBCL in eight patients (5.1%). Indolent Non-Hodgkin Lymphoma (2.5%, *n* = 8) and T-cell lymphoma (1.3%, *n* = 2) were only found in individual patients. A total of 71 patients were enclosed with unilocular lesions (45.2%), and 86 patients suffered from multilocular lesions (54.8%). The vast majority, 79.6%, of lesions were located supratentorially in patients; in comparison, 9.6% were infratentorial, and 17 patients (10.8%) showed lesions located supratentorially as well as infratentorially. In 65 patients, periventricular localisation of the target lesion was described (41.4%).

### 3.1. The Surgical Approach—Statistical Evaluation of Subgroups

The cohort was divided into subgroups relating to the surgical approach that was used.

**Figure 2:**
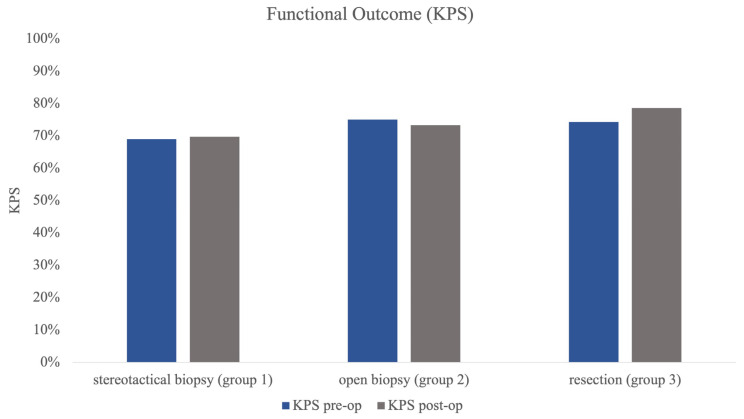
Figure 2 illustrating pre- and post-op KPS in all 3 subgroups as functional outcome measure. Statistical testing revealed no significant differences in pre- and post-op status.

Group 1 (stereotactical biopsy) was the largest group of all three subgroups (*n* = 101) with a mean age of 63.92 years (±SD 13.04). Mean number of lesions was 3.17 (±SD 2.60) with a mean volume of 8.5 mL (±SD 11.57) of the largest lesion. The mean KPS pre-op was 69.31% (±SD 15.18) with almost the same KPS post-op (69.70%, ±SD 15.97) (Figure 2).

Group 2 (open biopsy) was the smallest group with 21 patients. Mean age was higher than in group 1 at 69.86 years (±SD 10.83). Group 2 showed a mean number of lesions of 2.90 (±SD 2.39) and a mean largest volume of 12.88 mL (±SD 14.89). Pre-op mean KPS was higher, at 74.76% (±SD 18.87), and showed almost no change in postoperative evaluation (post-op KPS 73.33%, ±SD 20.33%, Figure 2).

The resection group (group 3) showed a mean age of 64.91 years (±SD 12.27) in 35 patients. Mean number of lesions was significantly smaller than in the other groups at 1.46 (±SD 0.82) (*p* < 0.001) lesions, and volumetry revealed the largest mean tumour volume of all groups at 21.15 mL (±SD 29.45), which was significantly larger than in groups 1 and 2 (*p* = 0.002). KPS post-op also improved significantly in comparison to the preoperative testing (pre-op 74.29% vs. post-op 78.57%, *p* = 0.021) in group 3 (Figure 2).

Supratentorial lesions were found in the majority in all groups (group 1 79.2%, *n* = 80; group 2 81%, *n* = 17; group 3 80%, *n* = 28). Periventricular lesions significantly more often were found in group 1 (52.5% vs. 42.9% group 2, vs. 8.6% group 3, *p* < 0.001).

### 3.2. Predicted Median Overall Survival by MSKCC Score

The majority of patients showed an MSKCC Score of 2 (66.2%, *n* = 104), predicting a median overall survival of 3.2 years according to the literature [16]. In nearly one quarter of patients (*n* = 38, 24.2%), scoring revealed the least favourable prognosis, with predicted median overall survival of only 1.1 years. Only 15 patients (9.6%) showed a favourable MSKCC Score of 1 (predicted median overall survival of 8.5 years). Additionally, subgroup analysis showed that most patients independently from their surgical approach had an MSKCC Score of 2. Chi-square test revealed no statistical significance concerning evaluation of MSKCC Score and surgical approach (*p* = 0.523).

### 3.3. Calculated Progression-Free and Overall Survival

Kaplan–Meier curves were calculated for illustration of PFS and OAS in different surgical approaches. Median follow-up was calculated as median time to censored and was 34.5 months.

Median PFS in the cohort was 5 months (95% CI 2.247–7.753), median OAS 12 months (95% CI 3.047–20.953).

**Figure 3:**
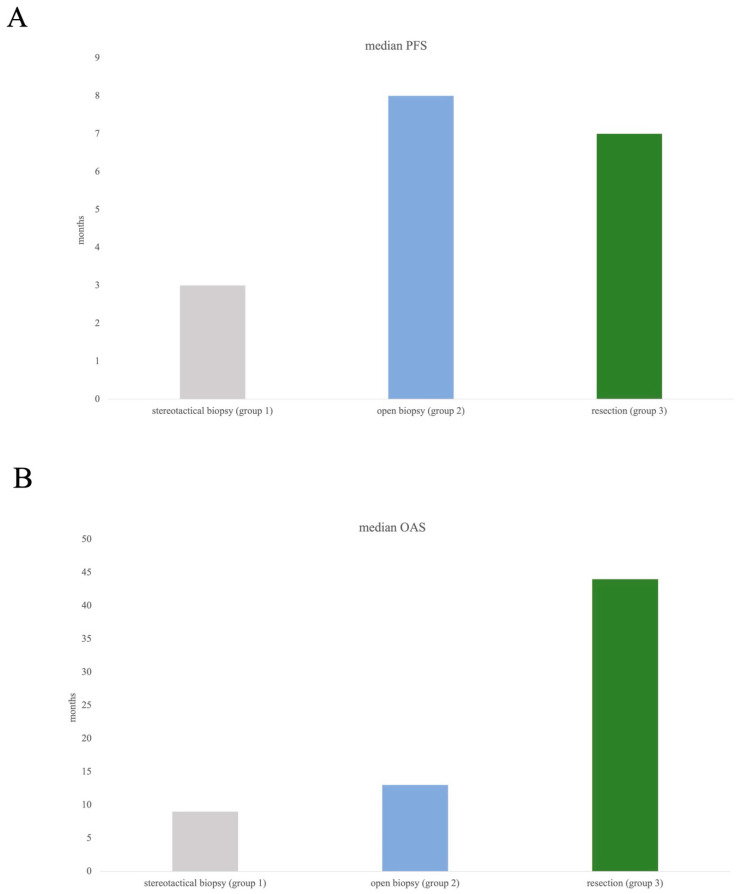
Median PFS (**A**) and median OAS (**B**) in all three subgroups. Log-rank test did not show any significance concerning comparison of three groups, which might have been influenced by the arguably small subgroups (resection *n*= 35, open biopsy *n*= 21).

According to subgroup evaluation, the longest median PFS was seen in group 2 (open biopsy) at 8 months, followed by in group 3 (resection) with a median PFS of 7. Median PFS in group 1 was 3 months (Figure 3A).

Overall survival showed most favourable data in group 3, with a median OAS of 44 months compared to 13 months (group 2) and 9 months (group 1), Figure 3B.

**Figure 4:**
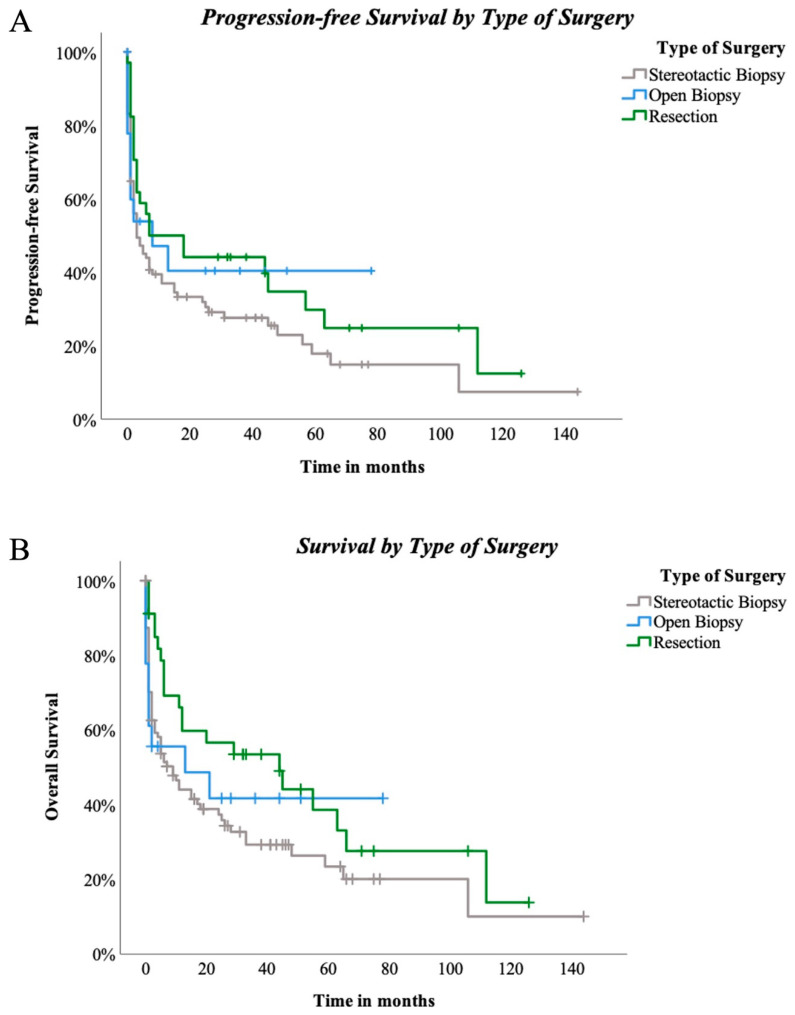
(**A**)—progression-free survival by type of surgery: PFS compared between stereotactic biopsy and open biopsy and resection in 147 patients. Log-rank (Mantel-Cox) *p* = 0.222. (**B**)—Survival by type of surgery: OS compared between stereotactic biopsy and open biopsy and resection in 147 patients. Log-rank (Mantel–Cox) *p* = 0.123.

**Figure 5:**
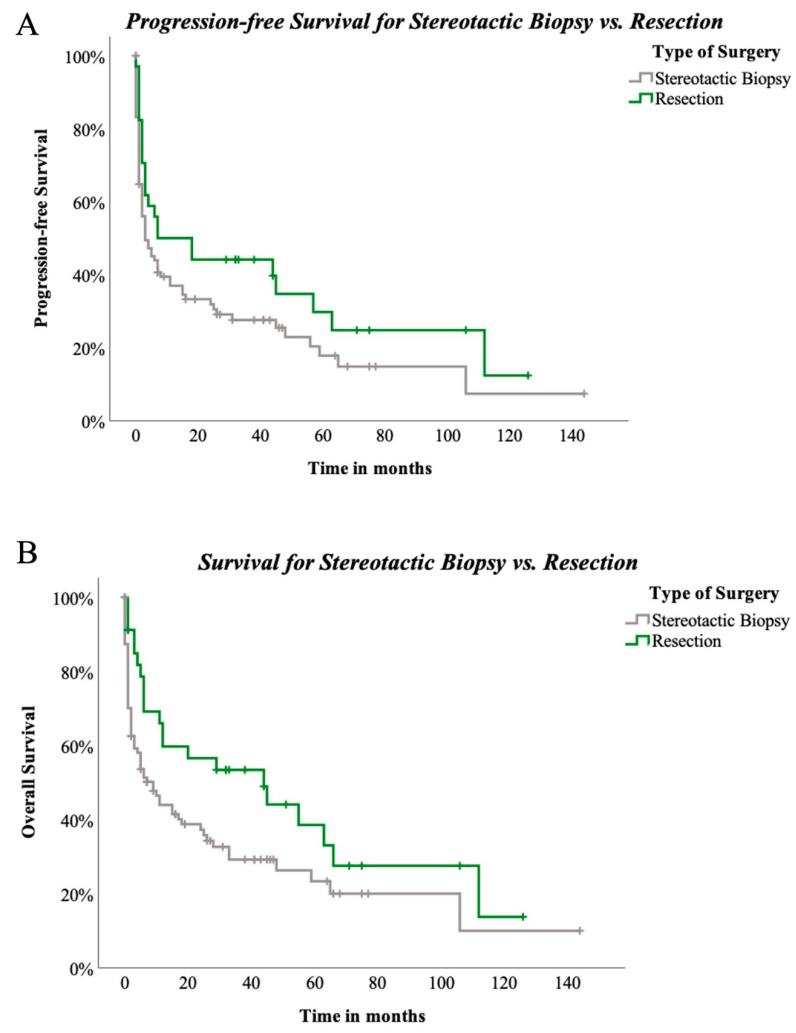
(**A**)—PFS compared between stereotactic biopsy and resection in 129 patients. Log-rank (Mantel–Cox) *p* = 0.089, (**B**)—OAS compared between stereotactic biopsy and resection in 129 patients. Log-rank (Mantel–Cox) *p* = 0.035.

Surgical techniques did not reveal significant differences for PFS among the three groups (*p* = 0.222, Figure 4A). OAS was also not significantly influenced by the three different types of surgical approach (*p* = 0.123, Figure 4B). Nevertheless, when stereotactical biopsy and resection were directly compared, positive trending regarding resection and PFS in comparison to stereotactical biopsy only was seen (*p* = 0.089, Figure 5A). Concerning OAS, results became significant in two-subgroup analysation (*p* = 0.035, Figure 5B).

### 3.4. Annual Survival Rates

**Figure 6:**
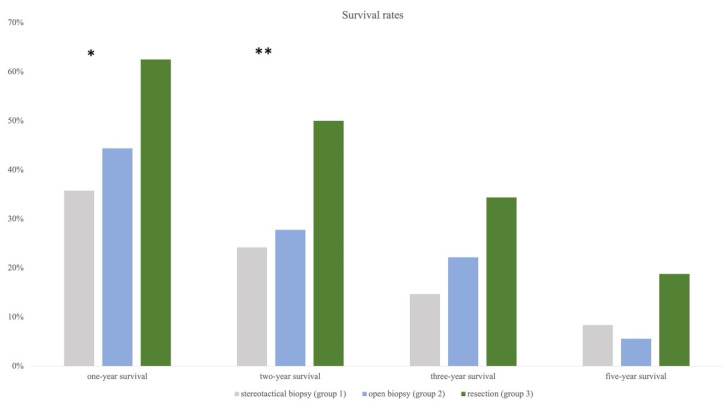
Annual survival rates showing significant results for resection compared to the other two groups after one and two years (* indicating significance). Statistical trend remains strong for three and five years even though significance was failed.

Based on OAS, survival rates at the time of 12 (one-year survival), 24 (two-year survival), 36 (three-year survival), and 60 months (five-year survival) were calculated for each group. One-year survival was highest in group 3 at 61.8%, followed by in groups 2 and 1 (44.4%, 36.8%). Chi-square test showed a significant difference between all groups (*p* = 0.042). The same trend was observed for two-year survival (group 3 = 52.9%, group 2 = 33.3%, group 1 = 27.4%) and also reached statistical significance (*p* = 0.026). Three-year and five-year survival rates were still highest in group 3, followed by in groups 2 and 1, except the chi-square test remained insignificant (Figure 6).

### 3.5. Risk Factors Influencing OS

We used the Cox regression model to identify risk factors for overall survival (OS) in our entire study cohort. In the initial univariate analysis, we examined the following variables: age in years, gender, MSKCC Score, presence of SCNSL, presence of multilocular lesions, number of lesions, type of surgery, pre- and postoperative Karnofsky Performance Status (KPS), lesion localisation (left, right, or bilateral hemispheres; supra-, infratentorial, or both; periventricular localisation), midline shift in millimetres, pre- and postoperative tumour volume of the largest lesion in cubic centimetres, and the presence of postoperative complications. Nominal variables were computed as categorial variables, with the first category referred to as indicator. Significant effect on OS was found for age (HR 1.038, 95% CI 1.018–1.059; *p* < 0.001), MSKCC Score (HR 1.553, 95% CI 1.085–2.225; *p* = 0.016), preoperative KPS (HR 0.984, 95% CI 0.973–0.995; *p* = 0.004), and post-op KPS (HR 0.976, 95% CI 0.965–0.987; *p* < 0.001). Regarding the type of surgery, resection was identified as the most influential predictor for longer OS compared to stereotactic biopsy (HR 0.609, 95% CI 0.370–1.001; *p* = 0.051), but this association did not reach statistical significance.

In the subsequent multivariate analysis, using a backward stepwise approach, we incorporated the significant predictors from the univariate analysis along with the type of surgery. First, preoperative KPS was excluded from the analysis (adjusted HR 1.005, 95% CI 0.980–1.030; *p* = 0.705). Second, the remaining variables were confirmed as predictors for OS. Age (adjusted HR 1.053, 95% CI 1.030–1.077; *p* < 0.001) emerged as the most substantial risk factor for shorter OS. Conversely, MSKCC Score (adjusted HR 0.593, 95% CI 0.360–0.975; *p* = 0.039), resection compared to stereotactic biopsy (adjusted HR 0.582, 95% CI 0.347–0.975; *p* = 0.040), and postoperative KPS (adjusted HR 0.967, 95% CI 0.953–0.982; *p* < 0.001) were associated with a reduced risk of shorter OS. Notably, a higher MSKCC Score correlated with a lower risk of shorter OS. Detailed results of the Cox regression analysis can be found in Table 2.

## 4. Discussion

As CNSL is a relatively rare tumour entity with no clear recommendation in favour of primary surgical resection, to date, there have been no prospective studies examining the effect of primary resection. Sometimes, patients undergo primary resection even when lymphoma is diagnosed later due to difficulty in differential diagnosis by MRI. In other cases, resection is performed due to emergency indications such as increased intracranial pressure with rapid neurological deterioration, but these cases remain infrequent. We aimed to find differences in clinical outcome and survival among patients who underwent a surgical resection with primary intent of cytoreduction compared to surgery such as stereotactical biopsy with diagnostic intent only.

### 4.1. Epidemiologic Factors

We could not find any significant difference in age at diagnosis between the analysed subgroups. Median age was around the 70th year of life. This underlines that CNSL is a disease of the elderly, whose life expectancy is already shortened [17]. Rising incidences of pCNSL for the elderly have been described over the last decades [18]. Taken this into consideration, one can assume that age and comorbidities have a strong influence on reported deaths. In our univariate and multivariate analysis, there was a clear correlation between higher age at diagnosis and shortened OS. These findings support a recently published large study comprising 539 patients with pCNSL. The authors of this study also found that increasing age at diagnosis and increasing Cooperative Oncology Group performance status (ECOG) was coherent with shorter OS, in line with our findings [19].

### 4.2. Tumour Classification

Most patients in our analysis suffered from pCNSL. The selection for type of surgery within patients with pCNSL and those with sCNSL seemed to be balanced as there was no statistically significant difference in distribution between the type of surgery groups (*p* = 0.729). The current literature mostly focuses on pCNSL; sCNSL mostly is excluded in analyses of surgical strategies. The therapy regimen usually concentrates on the primary lesion with extensive chemotherapy agents to reach the CNS [3]. Studies for evaluation of resection within a patient cohort of sCNSL are lacking at the moment. However, we identified seven patients with an sCNSL in the resection group. In the overall univariate analysis, presence of sCNSL could not be identified as a risk factor for reduced OS. Nevertheless, it still remains unclear whether patients with sCNSL benefit from resection. Concerning tumour histology, in accordance with epidemiologic findings [5], we found DLBCL as the most frequent neuropathological diagnosis. In our cohort, only a few cases deviated from DLBCL as histological subtype, which is typically in CNSL.

### 4.3. Functional Outcome Evaluation

KPS as a measurement tool for clinical status is widely used in clinical routine. We used the KPS to detect comprehensible improvement or deterioration of functionality pre- versus post-op. Improvement of KPS at discharge was interestingly only observed for patients after resection. In addition, we found that a higher post-op KPS was one of the most significant predictors for prolonged OS. Similar findings for the impact of post-op KPS on survival were reported by Schellekes et al. in 2021. In their study, they included only unilocular pCNSL. They reported an OS similar to ours with a difference between patients that underwent biopsy (median OAS 12.8 months) and those that underwent resection (median OS 30.7 months). Unless they could not find a statistical significance (log-rank test *p* = 0.095) for the type of surgery, patients with superficial lesions and a postoperative KPS > 70 were identified to benefit from resection in terms of OS. Patient’s age and resectability of a lesion not only influence the selection for resection but also influence OS in general [20].

In 2006, a new prognostic score for pCNSL was created by Abrey et al. at the Memorial Sloan Kettering Cancer Center (MSKCC) in New York. They related age at diagnosis and KPS at diagnosis as the main prognostic indicators in a scoring system [16]. Using that system for our cohort caused a selection bias for the subsequent analysis. The vast majority of patients were older than 50, leading to a very small number of patients in MSKCC category one (*n* = 15) and shifting the median MSKCC value to two and above.

The result of the univariate analysis implicates a higher risk for higher MSKCC Scores with HR 1.553 (95% CI 1.085–2.225, *p* = 0.016). In contrast to the literature, our multivariate analysis indicated an inverted correlation with higher MSKCC Score correlating with lower risk for shorter OS. The authors assume that this was caused by the median Score shift.

### 4.4. MRI Evaluation

The role of stereotactic biopsy in obtaining a neuropathological diagnosis remains pivotal as it directly informs subsequent therapeutic strategies. However, recent developments have introduced novel concepts utilising neuroimaging as a precise tool for distinguishing between pCNSL and Glioblastoma, as documented by Lin et al., 2017, and Kang et al., 2021 [21,22]. The decision to pursue surgical intervention without prior histological confirmation typically relies on the surgeon’s expertise and radiological assessments. Notably, multilocular lesion characteristics and deep-seated tumour locations frequently prompt a biopsy-oriented approach, foregoing immediate cytoreduction. In 2018, Jahr et al. identified deep brain localisation as a paramount prognostic factor, yet they did not observe a significant difference in OS and PFS between craniotomy and biopsy procedures. Their study, characterised by comprehensive follow-up and data quality, offers valuable insights. However, it is worth noting that the sample size of their series may not provide statistically robust evidence [11].

Our data support the preference for resection, particularly in those with single lesions as opposed to multilocular lesions. The prevalence of unilocular lesions was highest in the resection group, accounting for 74% of cases, compared to 48% for open biopsy and 35% for stereotactic biopsy (*p* < 0.001). This supports the authors’ impression that particularly patients with unilocular lesions and additionally inconclusive MRI with a KPS allowing surgical procedures might be the best candidates to profit from surgical therapy.

However, when we conducted a subgroup analysis focusing solely on patients with unilocular lesions, we did not observe a significant impact on OS or PFS based on the type of surgery performed (log-rank *p* = 0.324 and log-rank *p* = 0.316, respectively). It is important to acknowledge that the relatively small sample size of unilocular CNSL cases (*n* = 71) may have limited the statistical power of our findings. Nonetheless, a discernible statistical trend favouring resection was evident. Interestingly, none of the image morphological characteristics, including hemisphere localisation, supra- or infratentorial space involvement, periventricular localisation, presence of midline shift, pre- and postoperative tumour volume, or the occurrence of postoperative complications, emerged as prognostic risk factors in our Cox regression model. These results contrast with findings from previous studies by Schellekes and Jahr, which identified certain neuroimaging features as potential risk factors [11,20].

The existing literature on this subject matter now provides compelling evidence pointing towards a statistical trend in favour of extended OS and PFS in cases involving craniotomy with resection, as opposed to the purely diagnostic stereotactic biopsy procedure. Nonetheless, the process of patient selection for surgical resection remains a subject of contention as it is frequently subject to selection biases that complicate the discernment of the true impact of cytoreductive therapy on both survival outcomes and functional well-being. In our view, surgical resection should be regarded as an adjunct therapeutic strategy, in synergy with radiotherapy and chemotherapy. There is also evidence that intraoperative strategies that showed positive effects on extent of resection and OS in glioma patients, such as fluorescence-guided resection, might also have positive impacts in patients with CNSL [23]. Drawing parallels with the treatment of diffuse gliomas, cytoreduction can be conceptualised as the initial step in a standardised therapeutic regimen, which can subsequently be followed by established radiotherapy and chemotherapy protocols that have garnered recognition for their efficacy [8]. This perspective underscores the importance of a comprehensive and multidisciplinary approach to the management of central nervous system tumours, aiming to optimise patient outcomes.

### 4.5. Evaluation of Survival

In recent years, the impact of cytoreduction and the extent of resection on the survival of glioma patients have been extensively studied and documented [24,25,26,27] and might also be relevant for CNSL treatment. In the present cohort, the combination of primary resection with the variously evaluated and published protocols of combined immuno-/chemotherapy followed by ASCT [28,29,30,31,32] might have caused the observed improvement in one- and three-year survival rates. Preliminary findings suggest a potential enhancement of one- and three-year survival rates. Our study demonstrates a statistically significant trend in favour of resection over stereotactic or open biopsy as a primary intervention for CNSL.

A pivotal moment in re-evaluating the role of surgery in CNSL treatment occurred with the publication of a subsequent analysis of the G-PCNSL-SG-1 trial by Weller et al. in 2012. This trial challenged the long-standing recommendation against surgery, as it demonstrated significantly longer OS and PFS in patients who underwent gross total resection (GTR) or subtotal resection (STR) compared to in those who had a diagnostic biopsy only. A median OS of 32 months was reported in 67 cases for patients who underwent GTR and 31 months in 70 cases for those who had STR, compared to 18 months in 379 cases for biopsy-only patients. These findings were based on the largest PCNSL patient population studied to date, although a detailed neuroimaging review was lacking [12]. In 2018, Rae et al. compared three retrospective datasets, investigating the survival benefits of craniotomy over biopsy. Their study, encompassing a large cohort of patients, identified a correlation between craniotomy and extended overall survival in pCNSL. However, limitations in data heterogeneity and completeness warrant caution in interpreting these results [33]. Jiang et al. recently published data obtained in the context of the construction of a prognostic model for patient with pCNSL. Data of 2861 patients were included in the study, and the analysis revealed a significantly better OS in patients that underwent surgery with gross total resection compared to chemotherapy and/or radiotherapy alone [34].

In contrast to previous studies, our analysis included patients who underwent open biopsy solely for diagnostic purposes, involving craniotomy without intent for cytoreduction. This approach is reserved for specific cases where stereotactic biopsy may be inadequate due to preoperative steroid administration or diffuse tumour presentation on MRI scans. Recognising the complexity of such cases, we conducted a subgroup analysis excluding open biopsies. Kaplan–Meier analysis demonstrated a significant difference in OS between stereotactic biopsy and resection (log-rank *p* = 0.035), though PFS did not reach statistical significance (log-rank *p* = 0.089). This subgroup analysis offers a more nuanced perspective on outcomes, distinguishing between minimally invasive and open techniques with the goal of maximal cytoreduction. As for the safety of craniotomy and neurosurgical resection, previous studies linking invasiveness to adverse clinical outcomes can be dismissed given the substantial improvements in neurosurgical techniques over recent decades. Cloney et al. stated in 2017 that resection for pCNSL patients is safe, with similar complication rates as other intracranial tumours. However, they identified age, deep-seated lesions, and multilocular lesions as indicators for biopsy [13].

### 4.6. Limitations

Our study relied on retrospective data, a limitation that inherently constrains the robustness of our findings and introduces potential confounding biases. Unfortunately, our median follow-up period did not extend to median OS due to data gaps within our database. Notably, the distribution of patients across the three study groups was heavily skewed towards stereotactic biopsies. To attain statistical significance, it may be imperative to augment the sample size for the other groups. Additionally, it is important to acknowledge that the evaluation of neuroimaging data was conducted solely by one of the co-authors, without the validation of a neuroradiology consultant. A major limitation of the present study is the missing evaluation of adjuvant therapy strategies. We were able to gather data on adjuvant treatment from only a limited pool of 104 patients, representing 66% of the included patients. However, the collected data proved to be quite incomplete, largely because many patients either discontinued treatment or only underwent partial treatments (e.g., receiving radiation therapy without a comprehensive therapeutic plan) at the local University Hospital and continued treatment in external clinics. Additionally, the significant variation in treatment regimens, with detailed differences among them, resulted in a division of patients into multiple treatment groups, each with very small sample sizes. Consequently, a comprehensive statistical analysis of the obtained data was unfeasible. Due to these complexities, the authors decided not to provide further detailed explanations in this context. This recognition underscores the need for cautious interpretation of our findings and highlights areas for further refinement and validation in future research endeavours.

## 5. Conclusions

Our research aligns with the prevailing scientific consensus, highlighting a discernible trend toward improved survival outcomes and enhanced postoperative functionality. Notably, our findings differ from certain earlier publications in that we did not identify age as a significant selection bias influencing surgical decision-making. Despite the limitations of our analysis, our study lends further support to the notion of surgical resection serving as an additional therapeutic avenue for CNSL treatment. Nevertheless, it is imperative that we look towards prospective studies for a more comprehensive examination of this subject, free from inherent biases. In our perspective, surgical resection could play a more prominent role in the clinical decision-making process regarding the treatment of CNSL, representing an important avenue for further exploration.

## Figures and Tables

**Table 1:** Summary of cohorts’ general data and collected information on prognosis, functional outcome, and survival.

Variables	Neurosurgical Approach			*p*-Value
	Stereotactic Biopsy (*n* = 101)	Open Biopsy (*n* = 21)	Resection (*n* = 35)	
General				
mean age (years)	63.92 (13.04)	69.86 (10.83)	64.91 (12.27)	0.149 *
sex				0.732 **
female	53 (52.5%)	11 (52.4%)	21 (60%)	
male	48 (47.5%)	10 (47.6%)	14 (40%)	
classification				0.729 **
pCNSL	84 (83.2%)	16 (76.2%)	28 (80%)	
sCNSL	17 (16.8%)	5 (23.8%)	7 (20%)	
localization				<0.001 **
unilocular	35 (34.7%)	10 (47.6%)	26 (74.3%)	
multilocular	66 (65.3%)	11 (52.4%)	9 (25.7%)	
affected hemisphere				<0.001 **
right	16 (15.8%)	5 (23.8%)	19 (54.3%)	
left	24 (23.8%)	7 (33.3%)	12 (34.3%)	
both	61 (60.4%)	9 (42.9%)	4 (11.4%)	
periventricular lesions	53 (52.5%)	9 (42.9%)	3 (8.6%)	<0.001 **
mean number of lesions (±SD)	3.17 (2.60)	2.90 (2.39)	1.46 (0.82)	<0.001 *
tumour volume pre-op in cm^3^ (±SD)	8.53 (11.57)	12.88 (14.89)	21.15 (29.45)	0.002 *
tumour volume post-op in cm^3^ (±SD)	n.a.	n.a.	1.74 (2.87)	
Outcome & Survival				
MSKCC Score				0.523 **
1	11 (10.9%)	1 (4.8%)	3 (8.6%)	
2	62 (61.4%)	16 (76.2%)	26 (74.3%)	
3	28 (27.7%)	4 (19%)	6 (17.1%)	
mean KPS pre-op in % (±SD)	69.31 (15.18)	74.76 (18.87)	74.29 (16.85)	0.159 *
mean KPS post-op in % (±SD)	69.70 (15.97)	73.33 (20.33)	78.57 (13.75)	0.021 *
median OS in months (95% CI)	9 (*n* = 95) (3.894–14.106)	13 (*n* = 18) (0.000–45.533)	44 (*n* = 34) (8.184–79.816)	0.123 ***
median PFS in months (95% CI)	3 (*n* = 95) (0.000–6.377)	8 (*n* = 18) (0.000–22.852)	7 (*n* = 34) (0.000–24.143)	0.222 ***
survival rates in %				
1-year-survival	36.8%	44.4%	61.8%	0.042 **
2-year-survival	27.4%	33.3%	52.9%	0.026 **
3-year-survival	17.9%	22.2%	38.2%	0.054 **

* ANOVA; ** Chi-Square Test; *** Log-Rank Test.

**Table 2:** Results of Cox regression model for identification of risk factors concerning OAS in the cohort. Significant effects in the multivariate analysis on OAS was found for “age” (*p* < 0.001), with older age decreasing OAS. In contrast, MSKCC Score (0.039), resection vs. STX (*p* = 0.040), and postoperative functional outcome measured by KPS (*p* < 0.001) were identified as lowering risk factors.

Variable	Univariate Analysis		Multivariate Analysis	
	HR with 95% CI	*p*-Value	HR with 95% CI	*p*-Value
age in years	1.038 (1.018–1.059)	***<0.001***	1.053 (1.030–1.077)	***<0.001***
male sex	1.130 (0.753–1.696)	0.554		
MSKCC-Score	1.553 (1.085–2.225)	***0.016***	0.593 (0.360–0.975)	***0.039***
SCNSL	1.053 (0.614–1.806)	0.852		
multilocular	1.203 (0.804–1.802)	0.369		
number of lesions	1.035 (0.947–1.131)	0.453		
type of surgery		0.147		0.085
open biopsy vs. STX	0.885 (0.439–1.665)	0.645	0.605 (0.324–1.303)	0.224
resection vs. STX	0.609 (0.370–1.001)	0.051	0.582 (0.347–0.975)	***0.040***
preoperative KPS	0.984 (0.973–0.995)	***0.004***	1.005 (0.980–1.030)	0.705
postoperative KPS	0.976 (0.965–0.987)	***<0.001***	0.967 (0.953–0.982)	***<0.001***
hemisphere		0.858		
left vs. right	1.154 (0.671–1.984)	0.605		
both vs. right	1.120 (0.687–1.826)	0.649		
localization		0.590		
infratentorial vs. supratentorial	1.250 (0.677–2.308)	0.475		
both vs. supratentorial	0.784 (0.378–1.626)	0.513		
periventricular localization	1.129 (0.747–1.706)	0.564		
preoperative tumour volume of the largest lesion in cm^3^	0.996 (0.985–1.007)	0.516		
postoperative tumour volume of the largest lesion in cm^3^	0.998 (0.982–1.015)	0.857		
presence of postoperative complications	1.171 (0.674–2.035)	0.575		

## Data Availability

Data available on request due to privacy restrictions.

## References

[1] Ostrom Q.T., Patil N., Cioffi G., Waite K., Kruchko C., Barnholtz-Sloan J.S. (2020). CBTRUS Statistical Report: Primary Brain and Other Central Nervous System Tumors Diagnosed in the United States in 2013–2017. Neuro Oncol..

[2] Grommes C., DeAngelis L.M. (2017). Primary CNS Lymphoma. J. Clin. Oncol..

[3] Orellana-Noia V., Abousaud A. (2022). Secondary Central Nervous System Lymphoma: Updates in Treatment and Prophylaxis Strategies. Curr. Treat. Options Oncol..

[4] Alaggio R., Amador C., Anagnostopoulos I., Attygalle A.D., Araujo I.B.O., Berti E., Bhagat G., Borges A.M., Boyer D., Calaminici M. (2022). The 5th edition of the World Health Organization Classification of Haematolymphoid Tumours: Lymphoid Neoplasms. Leukemia.

[5] Giannini C., Dogan A., Salomão D.R. (2014). CNS lymphoma: A practical diagnostic approach. J. Neuropathol. Exp. Neurol..

[6] Sherman M.E., Erozan Y.S., Mann R.B., Kumar A.A., McArthur J.C., Royal W., Uematsu S., Nauta H.J. (1991). Stereotactic brain biopsy in the diagnosis of malignant lymphoma. Am. J. Clin. Pathol..

[7] Kanavaros P., Mikol J., Nemeth J., Galian A., Dupont B., Thiebaut J.B., Thurel C. (1990). Stereotactic biopsy diagnosis of primary non-Hodgkin’s lymphoma of the central nervous system. A histological and immunohistochemical study. Pathol. Res. Pract..

[8] Hoang-Xuan K., Deckert M., Ferreri A.J.M., Furtner J., Gallego Perez-Larraya J., Henriksson R., Hottinger A.F., Kasenda B., Lefranc F., Lossos A. (2023). European Association of Neuro-Oncology (EANO) guidelines for treatment of primary central nervous system lymphoma (PCNSL). Neuro Oncol..

[9] Gleissner B., Chamberlain M. (2007). Treatment of CNS dissemination in systemic lymphoma. J. Neurooncol..

[10] Calimeri T., Steidl C., Fiore P., Ferreri A.J.M. (2023). New hopes in relapsed refractory primary central nervous system lymphoma. Curr. Opin. Oncol..

[11] Jahr G., Da Broi M., Holte H., Beiske K., Meling T.R. (2018). The role of surgery in intracranial PCNSL. Neurosurg. Rev..

[12] Weller M., Martus P., Roth P., Thiel E., Korfel A. (2012). Surgery for primary CNS lymphoma? Challenging a paradigm. Neuro Oncol..

[13] Cloney M.B., Sonabend A.M., Yun J., Yang J., Iwamoto F., Singh S., Bhagat G., Canoll P., Zanazzi G., Bruce J.N. (2017). The safety of resection for primary central nervous system lymphoma: A single institution retrospective analysis. J. Neurooncol..

[14] Betensky R.A. (2015). Measures of follow-up in time-to-event studies: Why provide them and what should they be?. Clin. Trials.

[15] Jahr G., Broi M.D., Holte H., Beiske K., Meling T.R. (2018). Evaluation of Memorial Sloan-Kettering Cancer Center and International Extranodal Lymphoma Study Group prognostic scoring systems to predict Overall Survival in intracranial Primary CNS lymphoma. Brain Behav..

[16] Abrey L.E., Ben-Porat L., Panageas K.S., Yahalom J., Berkey B., Curran W., Schultz C., Leibel S., Nelson D., Mehta M. (2006). Primary central nervous system lymphoma: The Memorial Sloan-Kettering Cancer Center prognostic model. J. Clin. Oncol..

[17] Siegal T., Bairey O. (2019). Primary CNS Lymphoma in the Elderly: The Challenge. Acta Haematol..

[18] Mendez J.S., Ostrom Q.T., Gittleman H., Kruchko C., DeAngelis L.M., Barnholtz-Sloan J.S., Grommes C. (2018). The elderly left behind-changes in survival trends of primary central nervous system lymphoma over the past 4 decades. Neuro Oncol..

[19] David K.A., Sundaram S., Kim S.-H., Vaca R., Lin Y., Singer S., Malecek M.-K., Carter J., Zayac A., Kim M.S. (2023). Older patients with primary central nervous system lymphoma: Survival and prognostication across 20 U.S. cancer centers. Am. J. Hematol..

[20] Schellekes N., Barbotti A., Abramov Y., Sitt R., Di Meco F., Ram Z., Grossman R. (2021). Resection of primary central nervous system lymphoma: Impact of patient selection on overall survival. J. Neurosurg..

[21] Lin X., Lee M., Buck O., Woo K.M., Zhang Z., Hatzoglou V., Omuro A., Arevalo-Perez J., Thomas A.A., Huse J. (2017). Diagnostic Accuracy of T1-Weighted Dynamic Contrast-Enhanced-MRI and DWI-ADC for Differentiation of Glioblastoma and Primary CNS Lymphoma. AJNR Am. J. Neuroradiol..

[22] Kang K.M., Choi S.H., Chul-Kee P., Kim T.M., Park S.H., Lee J.H., Lee S.T., Hwang I., Yoo R.E., Yun T.J. (2021). Differentiation between glioblastoma and primary CNS lymphoma: Application of DCE-MRI parameters based on arterial input function obtained from DSC-MRI. Eur. Radiol..

[23] Evers G., Kamp M., Warneke N., Berdel W., Sabel M., Stummer W., Ewelt C. (2017). 5-Aminolaevulinic Acid-Induced Fluorescence in Primary Central Nervous System Lymphoma. World Neurosurg..

[24] Sanai N., Berger M.S. (2008). Glioma extent of resection and its impact on patient outcome. Neurosurgery.

[25] Sanai N., Polley M.Y., McDermott M.W., Parsa A.T., Berger M.S. (2011). An extent of resection threshold for newly diagnosed glioblastomas. J. Neurosurg..

[26] Duffau H. (2016). Long-term outcomes after supratotal resection of diffuse low-grade gliomas: A consecutive series with 11-year follow-up. Acta Neurochir..

[27] Kinslow C.J., Garton A.L.A., Rae A.I., Marcus L.P., Adams C.M., McKhann G.M., Sisti M.B., Connolly E.S., Bruce J.N., Neugut A.I. (2019). Extent of resection and survival for oligodendroglioma: A U.S. population-based study. J. Neurooncol..

[28] Houillier C., Dureau S., Taillandier L., Houot R., Chinot O., Moluçon-Chabrot C., Schmitt A., Gressin R., Choquet S., Damaj G. (2022). Radiotherapy or Autologous Stem-Cell Transplantation for Primary CNS Lymphoma in Patients Age 60 Years and Younger: Long-Term Results of the Randomized Phase II PRECIS Study. J. Clin. Oncol..

[29] Ferreri A.J.M., Cwynarski K., Pulczynski E., Fox C.P., Schorb E., Celico C., Falautano M., Nonis A., La Rosée P., Binder M. (2022). Long-term efficacy, safety and neurotolerability of MATRix regimen followed by autologous transplant in primary CNS lymphoma: 7-year results of the IELSG32 randomized trial. Leukemia.

[30] Ferreri A.J., Cwynarski K., Pulczynski E., Ponzoni M., Deckert M., Politi L.S., Torri V., Fox C.P., Rosée P.L., Schorb E. (2016). Chemoimmunotherapy with methotrexate, cytarabine, thiotepa, and rituximab (MATRix regimen) in patients with primary CNS lymphoma: Results of the first randomisation of the International Extranodal Lymphoma Study Group-32 (IELSG32) phase 2 trial. Lancet Haematol..

[31] Brezina T., von Dewitz H., Schroeder T., Ullrich S., Nachtkamp K., Reifenberger G., Malzkorn B., Sabel M., Haas R., Kobbe G. (2022). First-line high-dose therapy and autologous blood stem cell transplantation in patients with primary central nervous system non-Hodgkin lymphomas-a single-centre experience in 61 patients. Ann. Hematol..

[32] Schenone L., Alcantara M., Houillier C., Soussain C. (2023). First line treatments in primary central nervous system lymphomas in young patients. Curr. Opin. Oncol..

[33] Rae A.I., Mehta A., Cloney M., Kinslow C.J., Wang T.J.C., Bhagat G., Canoll P.D., Zanazzi G.J., Sisti M.B., Sheth S.A. (2019). Craniotomy and Survival for Primary Central Nervous System Lymphoma. Neurosurgery.

[34] Jiang Q., Zhan G., Jiang W., Xu Y., Zheng G., Jiang C., Lin D., Wang K., Zhu H. (2023). Prognostic model and treatment choices for patients with primary intracranial central nervous system lymphoma: A population-based study. Clin. Neurol. Neurosurg..

